# Bis(benzo-15-crown-5-κ^5^ *O*)strontium bis­(triiodide)

**DOI:** 10.1107/S1600536808012373

**Published:** 2008-04-30

**Authors:** Christine Walbaum, Ingo Pantenburg, Gerd Meyer

**Affiliations:** a Institut für Anorganische Chemie, Universität zu Köln, Greinstrasse 6, D-50939 Köln, Germany

## Abstract

The title compound, [Sr(C_14_H_20_O_5_)_2_](I_3_)_2_, obtained by slow evaporation of an ethanol/dichloro­methane solution (1:1) of SrCl_2_, benzo-15-crown-5 and I_2_, is built of sandwich-like [Sr(benzo-15-crown-5)_2_]^2+^ cations and isolated linear I_3_^−^ anions which are arranged in alternating layers parallel to (010). The triiodide anions are located in general positions, whereas the cations are located on centres of inversion.

## Related literature

For related literature, see: Pantenburg *et al.* (2002 ▶); Walbaum *et al.* (2007 ▶) and references cited therein. For bond-length data, see: Allen *et al.* (1987 ▶). For a description of the Cambridge Structural Database, see: Allen (2002 ▶).
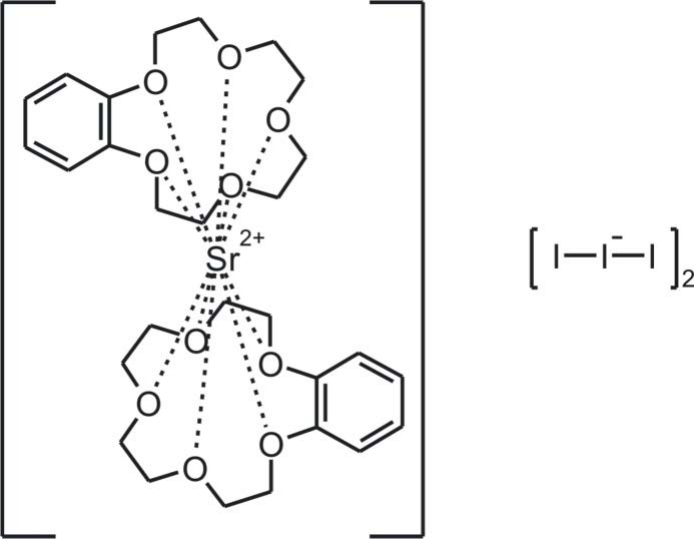


## Experimental

### 

#### Crystal data

[Sr(C_14_H_20_O_5_)_2_](I_3_)_2_
*M* *_r_* = 1385.62
Monoclinic, 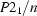

*a* = 12.0127 (17) Å
*b* = 12.8666 (12) Å
*c* = 13.085 (2) Å
β = 90.245 (18)°
*V* = 2022.5 (5) Å^3^
*Z* = 2
Mo *K*α radiation
μ = 5.96 mm^−1^
*T* = 293 (2) K
0.2 × 0.2 × 0.15 mm

#### Data collection

Stoe IPDS-I diffractometer
Absorption correction: numerical [*X-RED* (Stoe & Cie, 2001 ▶); after optimizing the crystal shape using *X-SHAPE* (Stoe & Cie, 1999 ▶)] *T*_min_ = 0.393, *T*_max_ = 0.465
18994 measured reflections
4869 independent reflections
1779 reflections with *I* > 2σ(*I*)
*R*_int_ = 0.124

#### Refinement

*R*[*F* ^2^ > 2σ(*F* ^2^)] = 0.040
*wR*(*F* ^2^) = 0.069
*S* = 0.71
4869 reflections
207 parameters
H-atom parameters constrained
Δρ_max_ = 0.54 e Å^−3^
Δρ_min_ = −0.72 e Å^−3^

### 

Data collection: *IPDS* (Stoe & Cie, 1996 ▶); cell refinement: *IPDS*; data reduction: *IPDS*; program(s) used to solve structure: *SIR92* (Altomare *et al*., 1993 ▶); program(s) used to refine structure: *SHELXL97* (Sheldrick, 2008 ▶); molecular graphics: *DIAMOND* (Brandenburg, 2004 ▶); software used to prepare material for publication: *CIF-Editor* (Wieczorrek, 2004 ▶).

## Supplementary Material

Crystal structure: contains datablocks global_, I. DOI: 10.1107/S1600536808012373/nc2102sup1.cif

Structure factors: contains datablocks I. DOI: 10.1107/S1600536808012373/nc2102Isup2.hkl

Additional supplementary materials:  crystallographic information; 3D view; checkCIF report

## Figures and Tables

**Table:** 

I2—I1	2.8754 (13)
I2—I3	2.9210 (13)
Sr1—O13	2.679 (5)
Sr1—O13^i^	2.679 (5)
Sr1—O4	2.682 (5)
Sr1—O4^i^	2.682 (5)
Sr1—O1	2.691 (5)
Sr1—O1^i^	2.691 (5)
Sr1—O7^i^	2.706 (5)
Sr1—O7	2.706 (5)
Sr1—O10^i^	2.778 (5)
Sr1—O10	2.778 (5)

**Table:** 

I1—I2—I3	177.54 (4)

Symmetry code: (i) 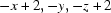.

**Fig. 1.**
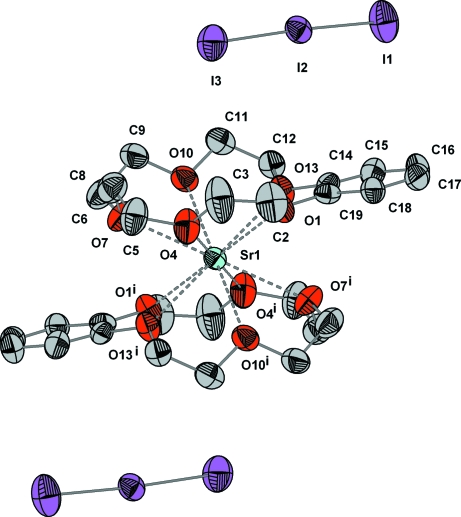
The structure of [Sr(benzo-15-crown-5)2](I3)2, showing the atom-numbering scheme and 50% probability displacement ellipsoids. Dashed lines denote Sr—O contacts. H atoms are omitted for clarity. Symmetry code: (i) = -x + 2, -y, -z + 2.

**Fig. 2.**
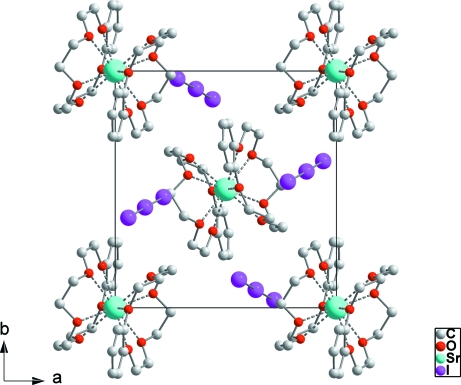
A projection of the structure of [Sr(benzo-15-crown-5)2](I3)2 along the ab plane. Hydrogen atoms are omitted for clarity.

## supplementary crystallographic information

### Comment

Polyiodide anions are synthesized by the addition of elemental iodine to iodide ions and can be incorporated into crystalline solids in the presence of suitable cations. They show considerable diversity in I - I bond lengths, covering the whole range between a strongly covalent bond and the sum of the van der Waals radii of two iodine atoms. However, the bond lengths are never uniform. As a consequence, the structural diversity of polyiodide ions is remarkably high. To date, no systematic procedure for the synthesis and crystallization of iodine-rich polyiodides is known, and this remains the ultimate goal of our work. We try to control the structures and composition of polyiodide matrices by variation of the shape, charge and size of the corresponding cations. In previous work, we have shown that bulky low-charged cations of the general formula [*M*(crown-ether)]*^x^*^+^ (where *M* is an element of group 1 or 2, or a rare earth-metal, crown-ether is benzo-18-crown-6, benzo-15-crown-5 or dibenzo-18-crown-6 and *x* = 1, 2 or 3) positively influence the stabilitity of polyiodides in the solid state (Walbaum *et al.*, 2007).

[Sr(benzo-15-crown-5)_2_](I_3_)_2_ is isotypic with the respective Ba compound (Pantenburg *et al.*, 2002). The Sr^2+^ ion (2a; 1,0,1) is located slightly above the centre of two benzo-15-crown ligands and coordinated in a sandwich-like manner by ten O atoms. The Sr—O distances vary between 2.679 (5) Å and 2.778 (5) Å (Table 1). Distances and angles within the crown-ether moiety are in good agreement with published data [mean values from the Cambridge Structural Database (Allen, 2002): CH_2_—O = 1.43 (3) Å, CH_2_—CH_2_ = 1.51 (2) Å, O—CH_2_—CH_2_ = 108.9 (13)°, CH_2_—O—CH_2_ = 111.4 (10)°]. The triiodide anion is almost symmetrical (I1—I2 = 2.8754 (13) Å, I2—I3 = 2.9210 (13) Å) and only slightly angular (I1—I2—I3 = 177.54 (4)°).

Although polyiodide ions tend to form even larger arrays through weak attractions between the anions, the shortest distance between the triiodide anions in [Sr(benzo-15-crown-5)_2_](I_3_)_2_ is 4.6623 (19) Å (I2—I1^i^; (i) = -*x* + 2, -*y* + 1, -*z* + 2) (Fig. 2). Thus, the anions may be considered as isolated. Distances between the [Sr(benzo-15-crown-5)_2_]^2+^ cations and the anions are also rather large, beginning with (I—H) = 3.138 (1) Å, (I—C) = 3.872 (9) Å, and (I—O) = 3.896 (5) Å.

### Experimental

[Sr(benzo-15-crown-5)_2_](I_3_)_2_ was prepared by dissolving SrCl_2_ (0.05 g, 0.3 mmol), C_14_H_20_O_5_ (0.08 g, 0.3 mmol), and I_2_ (0.08 g, 0.3 mmol) in ethanol/dichloromethane (1:1) (40 ml). Red crystals were obtained after a few days by slow evaporation of the solvent under ambient conditions.

### Refinement

The H atom were placed in idealized positions and constrained to ride on their parent atom, with C(ar)—H distances of 0.930 Å and *U*_iso_(H) values of 0.081 Å^2^ and C(al)—H distances of 0.970 Å and *U*_iso_(H) values of 0.092 Å^2^.

### Crystal data

**Table:**

[Sr(C_14_H_20_O_5_)_2_](I_3_)_2_	*F*_000_ = 1288
*M**_r_* = 1385.62	*D*_x_ = 2.275 Mg m^−^^3^
Monoclinic, *P*2_1_/*n*	Mo *K*α radiation λ = 0.71073 Å
Hall symbol: -P 2yn	Cell parameters from 1765 reflections
*a* = 12.0127 (17) Å	θ = 2.8–28.1º
*b* = 12.8666 (12) Å	µ = 5.96 mm^−^^1^
*c* = 13.085 (2) Å	*T* = 293 (2) K
β = 90.245 (18)º	Polyhedron, red
*V* = 2022.5 (5) Å^3^	0.2 × 0.2 × 0.15 mm
*Z* = 2	

### Data collection

**Table:**

Stoe IPDS-I diffractometer	4869 independent reflections
Radiation source: fine-focus sealed tube	1779 reflections with *I* > 2σ(*I*)
Monochromator: graphite	*R*_int_ = 0.124
Detector resolution: not measured pixels mm^-1^	θ_max_ = 28.1º
*T* = 293(2) K	θ_min_ = 2.8º
φ scans	*h* = −15→15
Absorption correction: numerical[X-RED (Stoe & Cie, 2001); after optimizing the crystal shape using X-SHAPE (Stoe & Cie, 1999)]	*k* = −16→17
*T*_min_ = 0.393, *T*_max_ = 0.465	*l* = −17→17
18994 measured reflections	

### Refinement

**Table:**

Refinement on *F*^2^	Secondary atom site location: difference Fourier map
Least-squares matrix: full	Hydrogen site location: inferred from neighbouring sites
*R*[*F*^2^ > 2σ(*F*^2^)] = 0.040	H-atom parameters constrained
*wR*(*F*^2^) = 0.069	*w* = 1/[σ^2^(*F*_o_^2^) + (0.0175*P*)^2^] where *P* = (*F*_o_^2^ + 2*F*_c_^2^)/3
*S* = 0.71	(Δ/σ)_max_ = 0.001
4869 reflections	Δρ_max_ = 0.54 e Å^−^^3^
207 parameters	Δρ_min_ = −0.72 e Å^−^^3^
Primary atom site location: structure-invariant direct methods	Extinction correction: none

### Special details

**Table:**

Experimental. A suitable single-crystal was carefully selected under a polarizing microscope and mounted in a glass capillary. The scattering intensities were collected on an imaging plate diffractometer (*IPDS* I, Stoe & Cie) equipped with a fine focus sealed tube X-ray source (Mo Kα, λ = 0.71073 Å) operating at 50 kV and 40 mA. Intensity data for the title compound were collected at room temperature by φ-scans in 100 frames (0 < φ < 200°, Δφ = 2°, exposure time of 7 min) in the 2 Θ range 3.8 to 56.3°. Structure solution and refinement were carried out using the programs *SIR92* (Altomare *et al.*, 1993) and *SHELXL97* (Sheldrick, 1997). A numerical absorption correction (*X-RED* (Stoe & Cie, 2001) was applied after optimization of the crystal shape (*X-SHAPE* (Stoe & Cie, 1999)). The last cycles of refinement included atomic positions for all atoms, anisotropic parameters for all non-hydrogen atoms and isotropic thermal parameters for all hydrogen atoms. The final difference maps were free of any chemically significant features. The refinement was based on F^2^ for ALL reflections.
Geometry. All e.s.d.'s (except the e.s.d. in the dihedral angle between two l.s. planes) are estimated using the full covariance matrix. The cell e.s.d.'s are taken into account individually in the estimation of e.s.d.'s in distances, angles and torsion angles; correlations between e.s.d.'s in cell parameters are only used when they are defined by crystal symmetry. An approximate (isotropic) treatment of cell e.s.d.'s is used for estimating e.s.d.'s involving l.s. planes.
Refinement. Refinement of *F*^2^ against ALL reflections. The weighted *R*-factor *wR* and goodness of fit *S* are based on *F*^2^, conventional *R*-factors *R* are based on *F*, with *F* set to zero for negative *F*^2^. The threshold expression of *F*^2^ > σ(*F*^2^) is used only for calculating *R*-factors(gt) *etc*. and is not relevant to the choice of reflections for refinement. *R*-factors based on *F*^2^ are statistically about twice as large as those based on *F*, and *R*- factors based on ALL data will be even larger.

### Fractional atomic coordinates and isotropic or equivalent isotropic displacement parameters (Å^2^)

**Table:**

	*x*	*y*	*z*	*U*_iso_*/*U*_eq_	
I2	1.13518 (5)	0.42408 (4)	1.15064 (4)	0.05056 (16)	
I3	1.05971 (6)	0.38042 (5)	1.35816 (5)	0.0757 (2)	
I1	1.21912 (7)	0.46748 (4)	0.94966 (5)	0.0820 (2)	
Sr1	1.0000	0.0000	1.0000	0.0349 (3)	
O10	1.1629 (5)	0.1317 (4)	1.0776 (4)	0.0519 (15)	
O4	1.1188 (5)	−0.1460 (4)	0.9031 (4)	0.0641 (17)	
O7	1.1714 (4)	0.0601 (4)	0.8803 (4)	0.0541 (15)	
O13	1.0602 (4)	−0.0068 (4)	1.1973 (3)	0.0508 (14)	
O1	1.0583 (5)	−0.1762 (3)	1.0971 (4)	0.0479 (14)	
C19	1.0374 (6)	−0.1842 (5)	1.1998 (6)	0.040 (2)	
C2	1.0947 (9)	−0.2637 (6)	1.0413 (7)	0.074 (3)	
H2A	1.1444	−0.3049	1.0836	0.092 (8)*	
H2B	1.0311	−0.3065	1.0234	0.092 (8)*	
C3	1.1501 (10)	−0.2336 (7)	0.9523 (8)	0.092 (4)	
H3A	1.2284	−0.2265	0.9693	0.092 (8)*	
H3B	1.1439	−0.2905	0.9040	0.092 (8)*	
C14	1.0380 (6)	−0.0936 (5)	1.2541 (6)	0.0406 (19)	
C9	1.2562 (8)	0.1596 (7)	1.0116 (7)	0.066 (3)	
H9A	1.2834	0.2283	1.0293	0.092 (8)*	
H9B	1.3166	0.1104	1.0210	0.092 (8)*	
C12	1.1048 (7)	0.0853 (5)	1.2453 (6)	0.048 (2)	
H12A	1.1309	0.0693	1.3138	0.092 (8)*	
H12B	1.0478	0.1385	1.2500	0.092 (8)*	
C11	1.1984 (7)	0.1221 (6)	1.1812 (6)	0.054 (2)	
H11A	1.2244	0.1889	1.2061	0.092 (8)*	
H11B	1.2597	0.0732	1.1856	0.092 (8)*	
C8	1.2194 (8)	0.1583 (6)	0.9073 (7)	0.064 (3)	
H8A	1.1646	0.2127	0.8969	0.092 (8)*	
H8B	1.2820	0.1724	0.8629	0.092 (8)*	
C6	1.2505 (7)	−0.0175 (7)	0.8513 (7)	0.065 (3)	
H6A	1.3005	−0.0323	0.9079	0.092 (8)*	
H6B	1.2944	0.0068	0.7940	0.092 (8)*	
C5	1.1885 (8)	−0.1121 (6)	0.8224 (7)	0.070 (3)	
H5A	1.1434	−0.0980	0.7623	0.092 (8)*	
H5B	1.2407	−0.1669	0.8054	0.092 (8)*	
C18	1.0111 (7)	−0.2778 (6)	1.2495 (6)	0.048 (2)	
H18	1.0087	−0.3400	1.2134	0.081 (15)*	
C15	1.0124 (7)	−0.0933 (6)	1.3557 (6)	0.056 (2)	
H15	1.0119	−0.0309	1.3915	0.081 (15)*	
C16	0.9875 (8)	−0.1844 (7)	1.4055 (7)	0.064 (3)	
H16	0.9698	−0.1837	1.4746	0.081 (15)*	
C17	0.9890 (7)	−0.2757 (7)	1.3526 (8)	0.063 (3)	
H17	0.9748	−0.3377	1.3868	0.081 (15)*	

### Atomic displacement parameters (Å^2^)

**Table:**

	*U*^11^	*U*^22^	*U*^33^	*U*^12^	*U*^13^	*U*^23^
I2	0.0578 (4)	0.0404 (3)	0.0535 (3)	−0.0024 (3)	−0.0036 (3)	−0.0060 (2)
I3	0.0831 (5)	0.0782 (4)	0.0659 (4)	−0.0055 (4)	0.0195 (4)	0.0070 (3)
I1	0.1280 (7)	0.0605 (4)	0.0576 (4)	0.0092 (4)	0.0162 (4)	0.0063 (3)
Sr1	0.0393 (7)	0.0292 (5)	0.0362 (6)	0.0017 (5)	−0.0024 (5)	0.0010 (4)
O10	0.058 (4)	0.048 (3)	0.049 (4)	−0.011 (3)	−0.013 (3)	0.003 (3)
O4	0.074 (5)	0.047 (3)	0.072 (4)	0.022 (3)	0.026 (4)	0.015 (3)
O7	0.045 (4)	0.046 (3)	0.072 (4)	−0.006 (3)	0.014 (3)	−0.007 (3)
O13	0.074 (4)	0.042 (3)	0.037 (3)	−0.012 (3)	−0.004 (3)	0.003 (2)
O1	0.070 (4)	0.037 (3)	0.037 (3)	0.009 (3)	0.001 (3)	−0.003 (2)
C19	0.035 (5)	0.038 (4)	0.048 (5)	0.005 (4)	−0.008 (4)	0.000 (4)
C2	0.108 (9)	0.035 (5)	0.078 (7)	0.023 (5)	0.022 (6)	−0.005 (5)
C3	0.138 (11)	0.064 (6)	0.074 (7)	0.023 (6)	0.043 (7)	0.006 (6)
C14	0.036 (5)	0.039 (5)	0.047 (5)	−0.009 (4)	−0.002 (4)	0.006 (4)
C9	0.059 (7)	0.075 (6)	0.064 (7)	−0.018 (5)	−0.020 (6)	0.020 (5)
C12	0.060 (6)	0.042 (4)	0.041 (5)	−0.009 (4)	−0.004 (4)	−0.007 (4)
C11	0.064 (7)	0.037 (4)	0.062 (6)	−0.009 (4)	−0.017 (5)	0.003 (4)
C8	0.053 (7)	0.056 (6)	0.084 (7)	−0.012 (5)	0.007 (6)	0.008 (5)
C6	0.058 (7)	0.073 (6)	0.064 (6)	0.000 (5)	0.010 (5)	−0.006 (5)
C5	0.079 (7)	0.060 (5)	0.071 (6)	0.005 (5)	0.032 (6)	−0.004 (5)
C18	0.051 (6)	0.041 (5)	0.052 (6)	−0.004 (4)	−0.002 (5)	0.010 (4)
C15	0.053 (6)	0.062 (6)	0.054 (6)	−0.008 (4)	0.007 (5)	0.005 (5)
C16	0.065 (7)	0.080 (7)	0.045 (6)	0.000 (5)	0.006 (5)	0.018 (5)
C17	0.049 (6)	0.062 (6)	0.078 (7)	0.002 (5)	0.000 (5)	0.037 (5)

### Geometric parameters (Å, °)

**Table:**

I2—I1	2.8754 (13)	C2—H2B	0.9700
I2—I3	2.9210 (13)	C3—H3A	0.9700
Sr1—O13	2.679 (5)	C3—H3B	0.9700
Sr1—O13^i^	2.679 (5)	C14—C15	1.365 (10)
Sr1—O4	2.682 (5)	C9—C8	1.434 (11)
Sr1—O4^i^	2.682 (5)	C9—H9A	0.9700
Sr1—O1	2.691 (5)	C9—H9B	0.9700
Sr1—O1^i^	2.691 (5)	C12—C11	1.483 (10)
Sr1—O7^i^	2.706 (5)	C12—H12A	0.9700
Sr1—O7	2.706 (5)	C12—H12B	0.9700
Sr1—O10^i^	2.778 (5)	C11—H11A	0.9700
Sr1—O10	2.778 (5)	C11—H11B	0.9700
O10—C11	1.424 (9)	C8—H8A	0.9700
O10—C9	1.463 (10)	C8—H8B	0.9700
O4—C3	1.350 (10)	C6—C5	1.475 (11)
O4—C5	1.418 (9)	C6—H6A	0.9700
O7—C6	1.431 (9)	C6—H6B	0.9700
O7—C8	1.431 (9)	C5—H5A	0.9700
O13—C14	1.369 (8)	C5—H5B	0.9700
O13—C12	1.443 (8)	C18—C17	1.377 (11)
O1—C19	1.371 (8)	C18—H18	0.9300
O1—C2	1.411 (8)	C15—C16	1.374 (10)
C19—C14	1.365 (9)	C15—H15	0.9300
C19—C18	1.405 (9)	C16—C17	1.364 (11)
C2—C3	1.399 (11)	C16—H16	0.9300
C2—H2A	0.9700	C17—H17	0.9300
			
I1—I2—I3	177.54 (4)	C3—C2—O1	111.1 (7)
O13—Sr1—O13^i^	180.0	C3—C2—H2A	109.4
O13—Sr1—O4	106.92 (17)	O1—C2—H2A	109.4
O13^i^—Sr1—O4	73.08 (17)	C3—C2—H2B	109.4
O13—Sr1—O4^i^	73.08 (17)	O1—C2—H2B	109.4
O13^i^—Sr1—O4^i^	106.92 (17)	H2A—C2—H2B	108.0
O4—Sr1—O4^i^	180.000 (1)	O4—C3—C2	119.7 (8)
O13—Sr1—O1	56.56 (14)	O4—C3—H3A	107.4
O13^i^—Sr1—O1	123.44 (15)	C2—C3—H3A	107.4
O4—Sr1—O1	59.66 (16)	O4—C3—H3B	107.4
O4^i^—Sr1—O1	120.34 (16)	C2—C3—H3B	107.4
O13—Sr1—O1^i^	123.44 (15)	H3A—C3—H3B	106.9
O13^i^—Sr1—O1^i^	56.56 (14)	C15—C14—C19	120.6 (7)
O4—Sr1—O1^i^	120.34 (16)	C15—C14—O13	124.8 (7)
O4^i^—Sr1—O1^i^	59.66 (16)	C19—C14—O13	114.5 (7)
O1—Sr1—O1^i^	180.000 (1)	C8—C9—O10	109.0 (7)
O13—Sr1—O7^i^	68.65 (16)	C8—C9—H9A	109.9
O13^i^—Sr1—O7^i^	111.35 (16)	O10—C9—H9A	109.9
O4—Sr1—O7^i^	118.78 (16)	C8—C9—H9B	109.9
O4^i^—Sr1—O7^i^	61.22 (16)	O10—C9—H9B	109.9
O1—Sr1—O7^i^	71.51 (16)	H9A—C9—H9B	108.3
O1^i^—Sr1—O7^i^	108.49 (16)	O13—C12—C11	107.3 (6)
O13—Sr1—O7	111.35 (16)	O13—C12—H12A	110.3
O13^i^—Sr1—O7	68.65 (16)	C11—C12—H12A	110.3
O4—Sr1—O7	61.22 (16)	O13—C12—H12B	110.3
O4^i^—Sr1—O7	118.78 (16)	C11—C12—H12B	110.3
O1—Sr1—O7	108.49 (16)	H12A—C12—H12B	108.5
O1^i^—Sr1—O7	71.51 (16)	O10—C11—C12	110.0 (7)
O7^i^—Sr1—O7	180.0	O10—C11—H11A	109.7
O13—Sr1—O10^i^	121.28 (15)	C12—C11—H11A	109.7
O13^i^—Sr1—O10^i^	58.72 (15)	O10—C11—H11B	109.7
O4—Sr1—O10^i^	77.00 (18)	C12—C11—H11B	109.7
O4^i^—Sr1—O10^i^	103.00 (18)	H11A—C11—H11B	108.2
O1—Sr1—O10^i^	80.80 (16)	O7—C8—C9	111.6 (7)
O1^i^—Sr1—O10^i^	99.20 (16)	O7—C8—H8A	109.3
O7^i^—Sr1—O10^i^	60.03 (17)	C9—C8—H8A	109.3
O7—Sr1—O10^i^	119.97 (17)	O7—C8—H8B	109.3
O13—Sr1—O10	58.72 (15)	C9—C8—H8B	109.3
O13^i^—Sr1—O10	121.28 (15)	H8A—C8—H8B	108.0
O4—Sr1—O10	103.00 (18)	O7—C6—C5	108.0 (7)
O4^i^—Sr1—O10	77.00 (18)	O7—C6—H6A	110.1
O1—Sr1—O10	99.20 (16)	C5—C6—H6A	110.1
O1^i^—Sr1—O10	80.80 (16)	O7—C6—H6B	110.1
O7^i^—Sr1—O10	119.97 (17)	C5—C6—H6B	110.1
O7—Sr1—O10	60.03 (17)	H6A—C6—H6B	108.4
O10^i^—Sr1—O10	180.00 (17)	O4—C5—C6	111.3 (7)
C11—O10—C9	110.9 (6)	O4—C5—H5A	109.4
C11—O10—Sr1	120.1 (4)	C6—C5—H5A	109.4
C9—O10—Sr1	118.3 (4)	O4—C5—H5B	109.4
C3—O4—C5	116.6 (7)	C6—C5—H5B	109.4
C3—O4—Sr1	120.4 (5)	H5A—C5—H5B	108.0
C5—O4—Sr1	116.9 (4)	C17—C18—C19	118.9 (8)
C6—O7—C8	114.5 (6)	C17—C18—H18	120.6
C6—O7—Sr1	117.5 (4)	C19—C18—H18	120.6
C8—O7—Sr1	114.6 (4)	C14—C15—C16	120.6 (8)
C14—O13—C12	120.4 (5)	C14—C15—H15	119.7
C14—O13—Sr1	119.8 (4)	C16—C15—H15	119.7
C12—O13—Sr1	119.4 (4)	C17—C16—C15	119.4 (8)
C19—O1—C2	120.4 (6)	C17—C16—H16	120.3
C19—O1—Sr1	118.5 (4)	C15—C16—H16	120.3
C2—O1—Sr1	120.5 (5)	C16—C17—C18	121.1 (8)
C14—C19—O1	116.5 (6)	C16—C17—H17	119.5
C14—C19—C18	119.4 (7)	C18—C17—H17	119.5
O1—C19—C18	124.1 (7)		

Symmetry codes: (i) −*x*+2, −*y*, −*z*+2.

## References

[bb11] Allen, F. H. (2002). *Acta Cryst.* B**58**, 380–388. 10.1107/s0108768102003890 12037359

[bb1] Allen, F. H., Kennard, O., Watson, D. G., Brammer, L. Orpen, A. G. & Taylor, R. (1987). *J. Chem. Soc. Perkin Trans. 2*, pp. S1–19.

[bb2] Altomare, A., Cascarano, G., Giacovazzo, C. & Guagliardi, A. (1993). *J. Appl. Cryst.* **26**, 343–350.

[bb3] Brandenburg, K. (2004). *DIAMOND* Bonn, Germany.

[bb4] Pantenburg, I., Hohn, F. & Tebbe, K.-F. (2002). *Z. Anorg. Allg. Chem.* **628**, 383–388.

[bb5] Sheldrick, G. M. (2008). *Acta Cryst.* A**64**, 112–122. 10.1107/S0108767307043930 18156677

[bb6] Stoe & Cie (1996). *IPDS* Stoe & Cie, Darmstadt, Germany.

[bb7] Stoe & Cie (1999). *X-SHAPE* Stoe & Cie, Darmstadt, Germany.

[bb8] Stoe & Cie (2001). *X-RED* Stoe & Cie, Darmstadt, Germany.

[bb9] Walbaum, C., Pantenburg, I. & Meyer, G. (2007). *Z. Anorg. Allg. Chem.* **633**, 1609–1617.

[bb10] Wieczorrek, C. (2004). *CIF-Editor* Universität zu Köln, Germany.

